# Expression analysis of miRNA hsa‐let7b‐5p in naso‐oropharyngeal swabs of COVID‐19 patients supports its role in regulating ACE2 and DPP4 receptors

**DOI:** 10.1111/jcmm.17492

**Published:** 2022-09-08

**Authors:** Andrea Latini, Chiara Vancheri, Francesca Amati, Elena Morini, Sandro Grelli, Matteucci Claudia, Petrone Vita, Vito Luigi Colona, Michela Murdocca, Massimo Andreoni, Vincenzo Malagnino, Massimiliano Raponi, Dario Cocciadiferro, Antonio Novelli, Paola Borgiani, Giuseppe Novelli

**Affiliations:** ^1^ Department of Biomedicine and Prevention, Genetics Unit University of Rome "Tor Vergata" Rome Italy; ^2^ Department of Experimental Medicine University of Rome "Tor Vergata" Rome Italy; ^3^ PTV Tor Vergata University Hospital Rome Italy; ^4^ Department of Systems Medicine University of Rome Tor Vergata Rome Italy; ^5^ Bambino Gesù Pediatric Hospital IRCCS Rome Italy; ^6^ Neuromed IRCCS Institute Pozzilli Italy; ^7^ School of Medicine Reno University of Nevada Reno Nevada USA

**Keywords:** ACE2, COVID‐19, DPP4, hsa‐let7b‐5p, miRNAs, SARS‐CoV‐2

## Abstract

Severe Acute Respiratory Syndrome Coronavirus‐2 (SARS‐CoV‐2) is the novel coronavirus responsible for worldwide coronavirus disease (COVID‐19). We previously observed that *Angiotensin‐converting enzyme 2 (ACE2)* and *Dipeptidyl peptidase‐4 (DPP4)* are significantly overexpressed in naso‐oropharyngeal swabs (NPS) of COVID‐19 patients, suggesting their putative functional role in the disease progression. *ACE2* and *DPP4* overexpression in COVID‐19 patients may be associated to epigenetic mechanism, such as miRNA differential expression. We investigated if hsa‐let7b‐5p, reported to target both *ACE2* and *DPP4* transcripts, could be involved in the regulation of these genes. We verified that the inhibition and overexpression of hsa‐let7b‐5p matched to a modulation of both *ACE2* and *DPP4* levels. Then, we observed a statistically significant downregulation (FC = −1.5; *p* < 0.05) of hsa‐let7b‐5p in the same COVID‐19 and control samples of our previous study. This is the first study that shows hsa‐let7b‐5p low expression in naso‐oropharyngeal swabs of COVID‐19 patients and demonstrates a functional role of this miR in regulating *ACE2* and *DPP4* levels. These data suggest the involvement of hsa‐let7b‐5p in the regulation of genes necessary for SARS‐CoV‐2 infections and its putative role as a therapeutic target for COVID‐19.

## 1 INTRODUCTION

Coronaviruses are a large family of viruses known to cause diseases ranging from common cold to more serious diseases such as Middle East Respiratory Syndrome (MERS) and Severe Acute Respiratory Syndrome (SARS).^1^ Among them, SARS‐CoV‐2 is the novel coronavirus responsible for worldwide COVID‐19.^2^ The clinical features of COVID‐19 are different, ranging from asymptomatic state or mild sub‐clinical symptoms to acute respiratory distress syndrome (ARSD) and to multi‐organ dysfunction according to the age, gender, and underlying patient health conditions.^3^ In addition, the clinical expression of COVID‐19 may also depend on the inter‐individual genetic variability that can make subjects more or less susceptible to the onset and progression of viral infection and therapy response.^4^, ^5^, ^6^, ^7^, ^8^

SARS‐CoV‐2 is a single‐stranded RNA (ssRNA) virus; following virus attachment and entry into host cells, the viral particle is uncoated and its positive‐sense ssRNA genome is released into citosol, where it serves as a matrix for the host translation machinery to produce viral proteins.^9^ This replication cycle potentially exposes viral RNA to an antiviral cellular defence arranged by the host's endogenous microRNAs (miRNAs). These miRNAs could directly degrade viral RNA and/or prevent viral protein translation, such as has been reported for the influenza virus.^10^ On the other end, to facilitate their infection, some viruses have developed strategies to exploit host miRNAs that regulate immunology tolerance.^11^, ^12^ Recently, an in silico approach identified potential host miRNAs that target genes involved in immune signalling pathways activated by SARS‐CoV‐2 infection.^13^, ^14^, ^15^ All these data suggest an important functional role of miRNAs in contrasting and/or taking part to viral infections.

Although miRNAs constitute only 3% of the human genome, they regulate approximately 60%–70% of coding genes, and alterations of their different expression levels are implicated in the development of several diseases, including viral diseases.^16^ Moreover, genes coding for miRNAs, as other genes, show genetic inter‐individual variability, and different studies have shown that genetic variants in miRNA genes can influence, in some cases, their expression, maturation, and even affinity with their target genes.^17^ Therefore, a possible variable that might be considered to influence the high clinical variability of COVID‐19 could also be the presence of these genetic variants, such as SNPs (single‐nucleotide polymorphisms) in microRNA target sites (MTSs) or miRNA sequences.^18^

In the last 2 years, several studies have investigated the potential function of microRNAs as biomarkers or therapeutic targets in COVID‐19, focusing primarily on circulating miRNA alterations.^19^ Although differences of miRNA expression profiles have been observed between infected and uninfected subjects,^20^ at present, little is known about possible miRNA expression changes in human nasopharyngeal tissue following SARS‐CoV‐2 infection.

Our previous paper described the significant upregulation of *Angiotensin‐converting enzyme 2 (ACE2)* and *Dipeptidyl peptidase‐4 (DPP4)* genes in naso‐oropharyngeal swabs (NPS) of COVID‐19 patients, thus suggesting that these receptors may play an important and complementary role in virus entry and in the onset and progression of disease.^21^ It has been widely reported that *ACE2*, highly expressed in the lungs and in particular in alveolar type II (AT2) cells, is the host receptor for SARS‐CoV‐2.^22^, ^23^, ^24^ *ACE2* is a type I integral membrane protein, involved in the regulation of the renin–angiotensin system and blood pressure, by the conversion of angiotensin II to the vasodilator Ang‐(1–7). *ACE2* is also involved in the regulation of several signalling pathways, including integrin signalling.^25^ However, *DPP4/CD26* has been reported as a co‐receptor of *ACE2*; in fact, SARS‐CoV‐2, such as MERS‐CoV, interacts with the identical residues of *DPP4*, in particular K267, R336, R317, and Q344.^26^, ^27^ DPP4 is a type II transmembrane glycoprotein, with serine exopeptidase activity, expressed in alveolar epithelial cells, endothelial cells, and in bronchiolar epithelial cells and involved in various biological processes as inflammation with post‐translational cleavage of hormones and chemokines, T‐cell activation, cell adhesion, and apoptosis.^27^ Moreover, *DPP4* had different roles in nutrition, metabolism and immune and endocrine systems.^28^

Literature data described several putative miRNAs targeting and regulating *ACE2* and *DPP4* genes.^29^, ^30^, ^31^, ^32^, ^33^

Among these, hsa‐let7b‐5p is a miRNA that potentially could target both *ACE2* and *DPP4* genes. Indeed, Zhang et al. have shown that hsa‐let7b‐5p promotes the development of hypoxic pulmonary hypertension by targeting ACE2.^30^ In addition, Srivastava et al. show that members of hsa‐let7 family, including hsa‐let7b‐5p, negatively regulate *DPP4* gene.^31^

Hsa‐let7b‐5p, one of the most studied members of hsa‐let7 miRNA family, has been extensively investigated in several diseases, such as cancer, cardiovascular diseases,^34^ and type 2 diabetes mellitus,^35^ and it is involved in inflammatory processes.^34^, ^36^, ^37^ Noteworthily, hsa‐let7b‐5p potentially could target SARS‐CoV‐2 in human bronchial epithelial cells according to a bioinformatics prediction.^38^ Moreover, hsa‐let7b‐5p also emerged among host miRNAs, identified by in silico analysis, that target genes involved in immune response pathways to SARS‐CoV‐2.^39^

*ACE2* and *DPP4* overexpression, observed in NPS of severe COVID‐19 patients,^21^ may also be linked to the regulatory effect of hsa‐let7b‐5p. The aim of this study is to investigate the putative modulation of *ACE2* and *DPP4* expression by hsa‐let7b‐5p and analyse its expression level in NPS of COVID‐19 patients.

## 2 MATERIALS AND METHODS

### 2.1 In silico prediction analysis

We performed an in silico analyses using the MiRWalk database (http://mirwalk.umm.uni‐heidelberg.de/) to identify the putative binding sites of hsa‐let7b‐5p on *DPP4* and *ACE2* transcripts. The MiRWalk database provides the largest available collection of miRNA–target interactions obtained from 12 established prediction programs. This database stores predicted data obtained with a machine‐learning algorithm including experimentally verified miRNA–target interactions.

### 2.2 Cell culture and miRNA overexpression and inhibition

HeLa cell line (ATCC) was cultured in complete medium DMEM supplemented with 10% Fetal Bovine Serum (FBS), 1X L‐glutamine at 37°C and 5% CO_2_. The cells were seeded at a 250,000 cell/well density and grown in complete culture medium. To overexpress and to inhibit the microRNA has‐let7b‐5p, HeLa cells were transiently transfected with mirVana® miRNA mimic and inhibitor, respectively (Thermofisher Scentistic). For both transfections, we used a final concentration of 45 pmol of mimic and inhibitor and 5 μl of Lipofectamine™ RNAiMAX Transfection Reagent (Invitrogen) following the manufacturer's instructions. Cells were harvested 24, 48 and 72 h after transfection and suspended in 500 μl of TRIzol (Ambion) until RNA extraction.

### 2.3 Expression study on transfected HeLa cells

Total RNA of transfected HeLa cells was extracted using TRIzol reagent (Ambion) according to the manufacturer’s instructions. One μg of total RNA was retrotranscribed in cDNA using the High Capacity cDNA Reverse Transcription Kit (Thermofisher Scientific) according to the manufacturer’s instructions. To evaluate the expression level of *ACE2* and *DPP4*, a qRT‐PCR (SYBR Green assay Applied Biosystems™) assay was performed, using Real‐Time PCR 7500 Software (Applied Biosystems™). Primers, qRT‐PCr conditions, and data analysis have been performed as described in our previous paper.^21^ *GADPH* has been selected as the housekeeping gene for data normalization in HeLa cells.

For the hsa‐let7b‐5p expression analysis, 500 ng of total RNA was retrotranscribed into cDNA using the miScript II RT kit (QIAGEN). To evaluate the hsa‐let7b‐5p expression level, ABI7500 Fast Real‐time PCR System (Applied Biosystems™ Themofisher Scientific), miScript SYBR Green PCR kit (QIAGEN), and miRNA‐specific miScript Primer Assays (hsa‐let7b‐5p, MS00003122) were performed. SNORD95 (MS00033726) was used for data normalization since it was referenced as housekeeping by different expression studies^40^, ^41^, ^42^; additionally, we did not observe any significant differences in its expression among our samples. The qRT‐PCR expression analyses were performed in duplicate. Data analysis was performed using the comparative threshold cycle (Ct) method quantification (2^‐ΔCt^ method) (https://www.protocols.io/view/comparative‐ct‐method‐quantification‐2‐ct‐method‐zp7f5rn).

### 2.4 Patients' recruitment

Sixty naso‐oropharyngeal swabs, derived from a cohort of COVID‐19 patients and SARS‐CoV‐2 negative subjects enrolled from 20th March to 20th April 2020, during the first severe pandemic wave in Italy, were collected to analyse miRNA expression. A venous blood sample (2 ml BD Vacutainer® EDTA Tubes) of all 60 subjects was also collected during their transition to the Tor Vergata University Hospital's, to sequence the *LET7b* gene. Available clinical data and SARS‐CoV‐2 host gene expression levels were extrapolated from previous analysis as already described in our previous paper.^21^

This study was approved by the ethical committee of Tor Vergata University Hospital ‐ Rome, Italy (protocol no. 50/20), and all patients received and signed a written informed consent, in agreement with the principles of the Declaration of Helsinki.

### 2.5 Diagnostic test of SARS‐CoV‐2

To detect the qualitative presence of SARS‐CoV‐2 viral nucleic acids in naso‐oropharyngeal swabs, we used Allplex™ 2019‐nCoV Assay (Seegene Inc.) (http://www.seegene.com/upload/product/Allplex_2019_nCoV_performance_data.pdf).^21^ A multiplex qRT‐PCR reaction targeting SARS‐CoV‐2 envelope (E) Nucleoprotein (N), and RNA‐directed RNA polymerase 1 (RdRP1) genes was used to diagnose acute viral infection by presence of viral RNA. Participants were diagnosed as COVID‐19‐positive with a comparative threshold cycle (Ct) value <40.

### 2.6 Hsa‐let7b‐5p expression study in naso‐oropharyngeal swabs

The residual swabs, obtained after viral RNA detection, contain both human RNA and microRNAs. RNA in residual swabs was evaluated by NanoDrop DS‐11 (DeNovix), and to isolate miRNA fraction, 50 ng of total RNA was retrotranscribed into cDNA using the miScript II RT kit (QIAGEN). The qRT‐PCR expression analyses were performed as described in paragraphs 2.3.

### 2.7 Genomic analysis

Genomic DNA of all 60 participants was extracted from 2 ml of peripheral blood samples using standard procedures and the Qiagen blood DNA mini‐Kit (Qiagen). *LET7B* gene was amplified by PCR and analysed by direct sequencing with ABI 3130xl Automated Sequencer (Applied Biosystems™). The amplified region included the pre‐miR region, which comprises both hsa‐let7b‐5p and hsa‐let7b‐3p strands, plus 200 bp upstream and downstream of the flanking sequence. Primers sequences are the following: 5′‐AGCCAGGGACTTCCCAAGA‐3′ and 5′‐AGTCTCATGACCTGGAACAG‐3′.

### 2.8 Statistical analysis

Statistical analyses of expression data were performed using GraphPad Prism 6.0 (GraphPad Software) and SPSS program, version 19 (IBM Corp). The Kolmogorov–Smirnov test was used to analyse the distribution of expression data from qRT‐PCR assays. The Mann–Whitney test, Kruskal‐Wallis test and anova test were used for data analysis as appropriate. Non‐parametric distribution, expression data, and clinical data are represented as mean and standard deviation (SD). For all analysis, significance was set at *p* ≤ 0.05.

## 3 RESULTS

### 3.1 In silico prediction analysis results

In order to confirm the interaction between hsa‐let7b‐5p and its two putative target genes, we first performed in silico analyses. The bioinformatic analysis through the prediction tool MiRWalk highlighted putative target sites of hsa‐let7b‐5p onto *DPP4* and *ACE2* transcripts. In particular, for the *ACE2* gene, we observed five putative binding sites (High Score = 1) in the two main transcripts (NM_021804 and NM_001371415), while for the *DPP4* gene, we found one putative binding site (Score = 0.85) (Table S1). All these putative binding sites are located on the coding region of *ACE2* and *DPP4* genes.

### 3.2 ACE2 and DPP4 modulation by hsa‐let7b‐5p mimic/inhibitor

To evaluate the regulatory effect of hsa‐let7b‐5p on the two putative gene targets, we performed in vitro functional assays. Hsa‐let7b‐5p mimics and inhibitors were used to functionally investigate the role of this miR on *ACE2* and *DPP4* expression levels.

**FIGURE 1:**
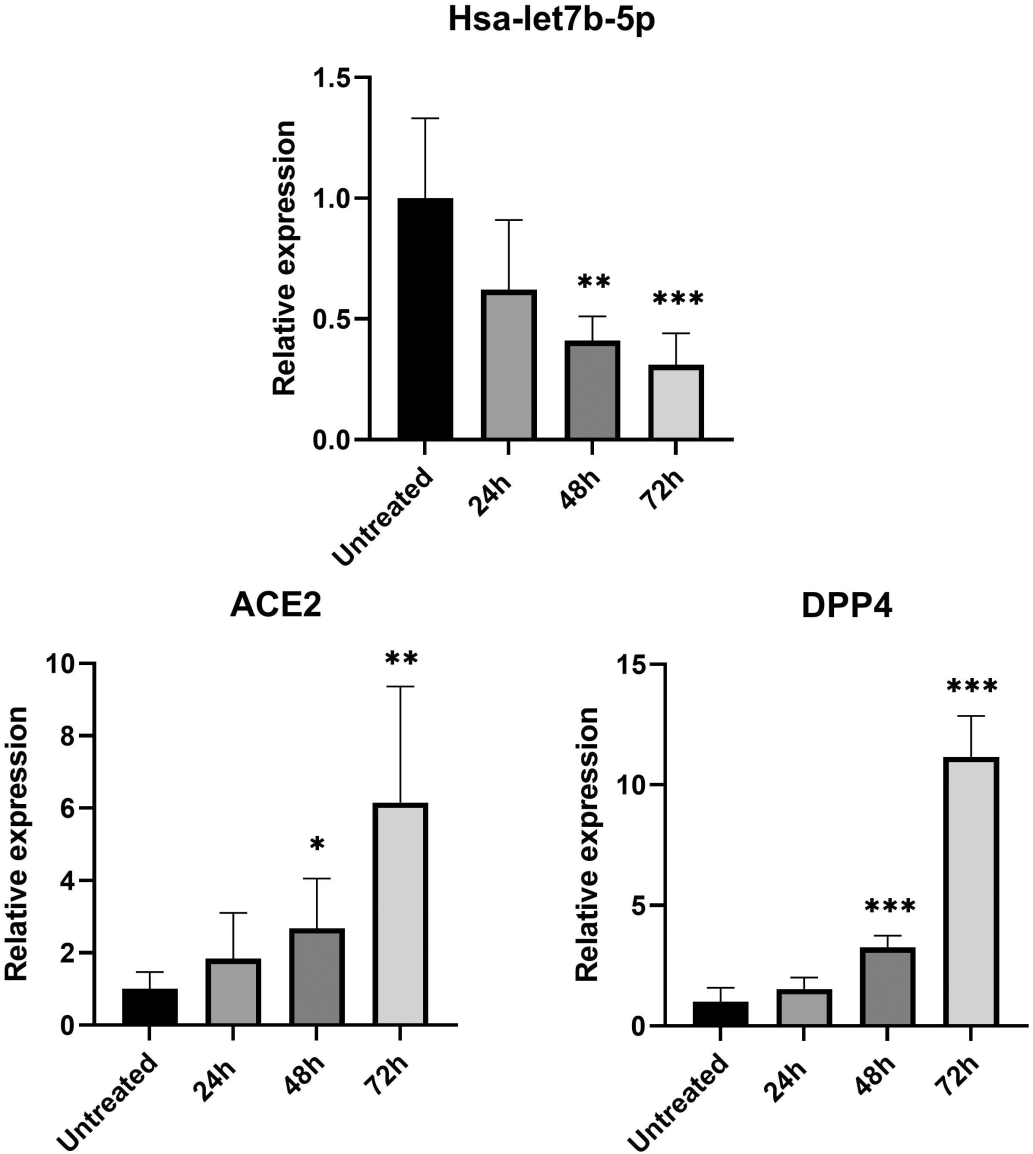
Effect of hsa‐let7b‐5p inhibition on ACE2 and DPP4 mRNA levels in HeLa cell line. Expression level of hsa‐let7b‐5p, ACE2, and DPP4 at three different time point (24, 48 and 72 h) after let7b‐5p inhibitor treatment. anova test was used for data analysis. Expression data (2‐ΔCt) are represented as mean with SD. *p‐value < 0.05, **p‐value < 0.01, ***p‐value < 0.001

**FIGURE 2:**
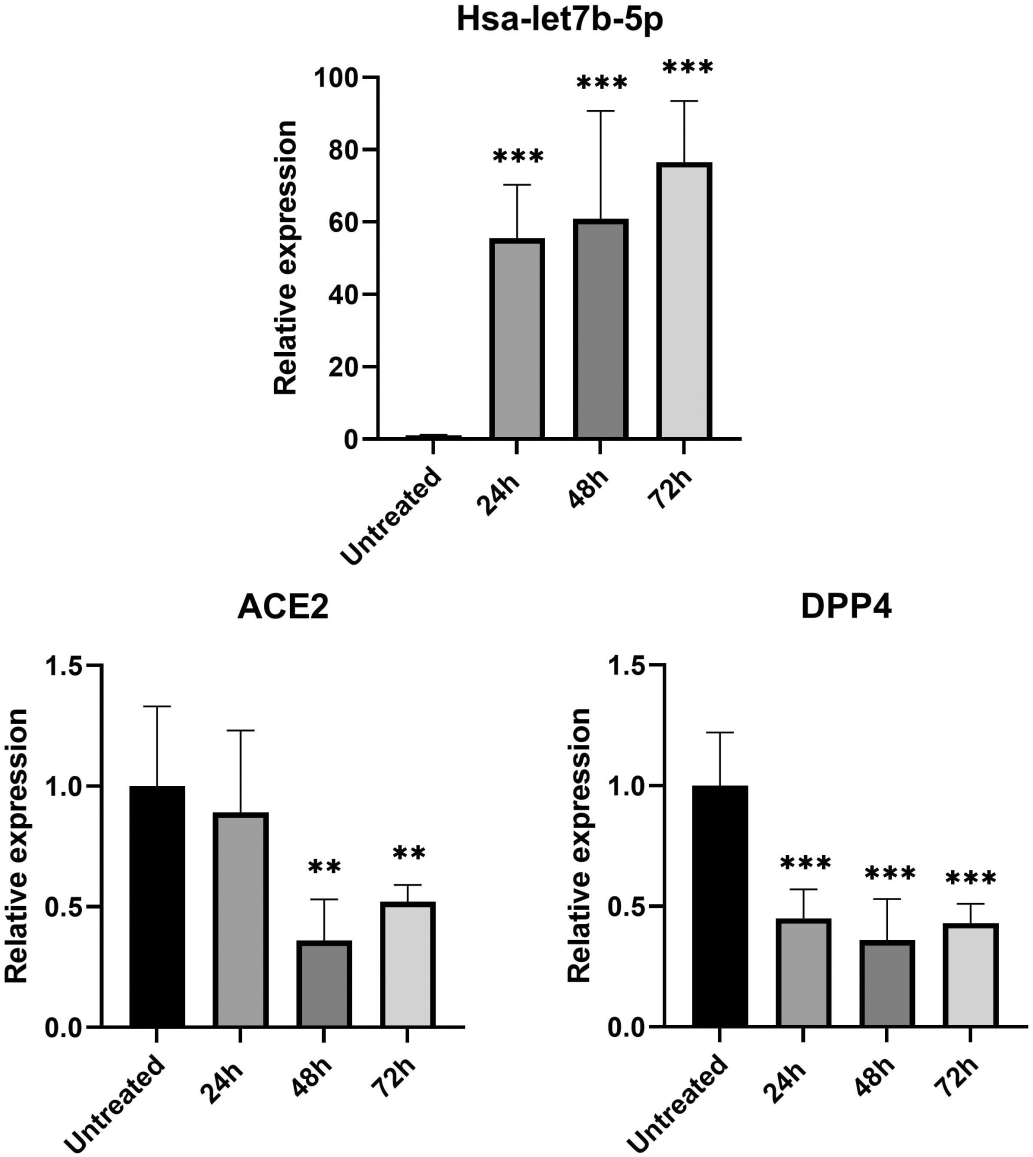
Effect of hsa‐let7b‐5p overexpression on ACE2 and DPP4 mRNA levels in HeLa cell line. Expression level of hsa‐let7b‐5p, ACE2, and DPP4 at three different time point (24, 48 and 72 h) after let7b‐5p mimic treatment. anova test was used for data analysis. Expression data (2^‐ΔCt^) are represented as mean with SD. **p‐value < 0.01, ***p‐value < 0.001.

As expected, in HeLa cells treated with the hsa‐let7b‐5p inhibitor, we observed a progressive increase in *ACE2* and *DPP4* mRNA levels compared to untreated cells (Figure 1). These changes of expression levels reached statistical significance after 48 h of treatment for both genes. In particular, hsa‐let7b‐5p inhibition induces a 6‐fold and 11‐fold increase in *ACE2* and *DPP4* expression, respectively. Accordingly, overexpression of hsa‐let7b‐5p in HeLa cells causes a significant decrease of *ACE2* mRNA levels compared to untreated cells, both at 48 and at 72 h after treatment. *DPP4* mRNA levels are significant decreased at all time points after treatment (Figure 2).

### 3.3 Sample collection

The 60 naso‐pharyngeal swabs (NPS) studied were collected at Tor Vergata University Hospital ‐ Rome, during the first lockdown period in Italy (20rd March–20th April 2020). Thirty‐five NPS derived from patients who showed symptoms (dry cough, fever, and dyspnea) referable to COVID‐19 and then resulted positive to the SARS‐CoV‐2 molecular test (COVID‐19 patients); the others NPS (n = 25) were from subjects who had contact with SARS‐CoV‐2 positive patients but resulted negative to the test and showed no clinical symptoms of COVID‐19 (Negative subjects). All COVID‐19 patients (*n* = 35) were hospitalized at Tor Vergata University Hospital. Available clinical data of the COVID‐19 patients are summarized and reported in Table S2 and in reference 21. The positive patients and negative subjects (controls) were matched for sex and age.

Most of the enrolled COVID‐19 patients were male (M = 26/35; 75%). Age ranged between 20 and 92 years (mean age ± SD = 62 ± 16; Men mean age ± SD = 59 ± 16; Female mean age ± SD = 66 ± 17). Age in SARS‐CoV‐2 negative group (*n* = 25, control group) ranged from 27 and 84 years old (mean age ± SD = 58 ± 16): nineteen out of 25 were men (76%; mean age ± SD = 58 ± 16), and six out of 25 were women (24%; mean age ± SD = 59 ± 21).

### 3.4 Hsa‐let7b‐5p expression level in SARS‐CoV‐2 positive and negative naso‐oropharyngeal swabs

**FIGURE 3:**
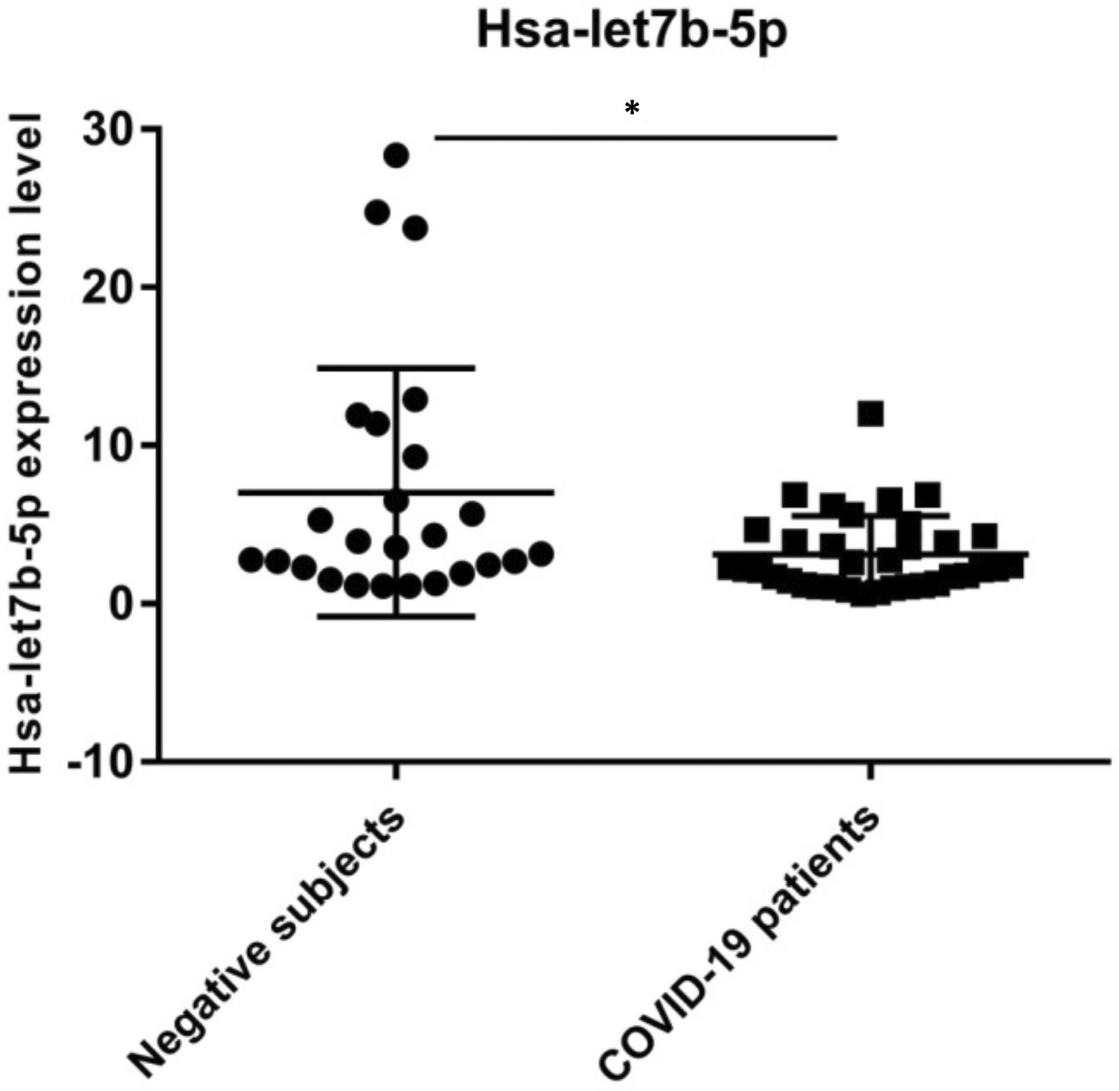
Hsa‐let7b‐5p expression level in Negative subjects vs COVID‐19 patients (*p*‐value<0.05). The Kolmogorov–Smirnov test was used to analyse the distribution of expression data. The Mann–Whitney test was used for data analysis. Expression data (2^‐ΔCt^) are represented as mean with SD.*p < 0.05.

We analysed the expression level of hsa‐let7b‐5p miRNA in the 60 naso‐oropharyngeal residual swabs described above and previously investigated for *ACE2* and *DPP4* expression.^21^ In this study, we detected an overexpression of *ACE2* and *DPP4* genes in the 35 severe COVID‐19 patients. Hsa‐let7b‐5p displayed a significant differential expression in NPS (*p*‐value<0.05) (Figure 3); in fact, hsa‐let7b‐5p resulted in downregulation in COVID‐19 patients compared to negative subjects. The decrease of hsa‐let7b‐5p expression levels in NPS of COVID‐19 patients is in line with the overexpression of *ACE2* and *DPP4* genes (*ACE2* FC = +1.88, *p* ≤ 0.05; *DPP4* FC = +3, *p* < 0.01) in the same patient samples, observed in our previous paper.^21^

### 3.5 Genomic analysis

In order to verify if the presence of genetic variants might associate with different hsa‐let7b‐5p expression levels, we sequenced the *LET7B* gene in all 60 enrolled subjects. After direct sequencing of pre‐miR region plus 200 bp upstream and downstream, we identified only one single‐nucleotide polymorphism (SNP) in the *LET7B* gene: rs143676966 (G > C). We did not observe significant difference in the variant allele frequency of this polymorphism between positive (2.2%) and negative (5.5%) patients. Moreover, the observed frequencies are similar to the frequency reported in the GnomAD database for non‐Finnish Europeans (2.2%). No statistically significant association was observed between the rs143676966 polymorphism and the expression level of hsa‐let7b‐5p.

### 3.6 Correlation analysis

We analysed, by Pearson correlation test, the relationship among the expression profile of the hsa‐let7b‐5p and ACE2 and DPP4 transcripts, respectively in COVID‐19 patients (*n* = 35) and negative subjects (*n* = 25). We observed a negative correlation between hsa‐let7b‐5p and ACE2 and DPP4 expression level (Pearson *r* = −0.215 and −0.200, respectively), although it does not reach statistical significance. Moreover, we verified whether the expression levels of hsa‐let7‐5p was correlated with any inflammatory markers, such as IL‐6, TNF‐α, fibrinogen, and D‐dimer and with specific diseases symptoms, such as fever, dry cough, dyspnea, and gastrointestinal disorders. No correlation was found among the hsa‐let7b‐5p expression data and the clinical data of COVID‐19 patients.

## 4 DISCUSSION AND CONCLUSION

The pandemic coronavirus disease 2019, due to SARS‐CoV‐2 infection, has shown several differences in clinical manifestations and complications, suggesting variability in the disease onset and progression.^44^, ^45^ Age and chronic medical conditions are the most common COVID‐19 adverse prognostic factors; however, genetic variants in human genome can cause inter‐individual variation in disease susceptibility and/or severity of clinical features in COVID‐19.^4^, ^5^, ^43^, ^44^, ^45^, ^46^ A better knowledge of the host regulatory mechanisms opposing to SARS‐CoV‐2 virus infection and virulence can provide actionable insights to identify novel therapeutics against COVID‐19.

It is well known that miRNA expression profiles are cell‐type‐specific and can be affected, for example, by cellular stress responses such as endoplasmic reticulum stress and the downstream activation of stress responses.^47^, ^48^ Thus, the individual and epigenetic differences in the miRNA profiles during an infection in different cell types, such as naso‐oropharyngeal cells, could affect the effectiveness of the antiviral responses and disease severity.^38^ Viruses tend to increase their gene expression downregulating the host gene expression both at transcriptional and post‐transcriptional level.^49^ By this perspective, miRNAs are important master regulators controlling gene expression at the post‐transcriptional levels. Noteworthily, the expression level of miRNAs is generally altered in normal versus diseased conditions. Several studies performed in the peripheral blood of patients with COVID‐19 compared with the healthy controls have observed differences in miRNA profiles, and cluster analysis has revealed that many of the differentially expressed miRNAs target genes as peptidases, protein kinases, and protein of the ubiquitin system.^49^, ^50^ Moreover, computational predictions of direct interactions between miRNAs and human coronavirus RNAs and bioinformatic analysis of binding sites have identified several host miRNAs with high binding probability to this virus family.^39^, ^51^ All these data bolster the innovative therapeutic approach that uses synthetic miRNAs (or alternatively siRNAs) to target and suppress specific translation of proteins critical for SARS‐CoV‐2 production and/or infection.^52^ For example, recent studies have tested exosomes, inorganic nanoparticles, and lipid moieties containing a mix of miRNAs that bind different regions on SARS‐CoV‐2 open‐reading frame (ORF) and 3’UTRs.^53^, ^54^

Since ACE2 appears to be the primary receptor for SARS‐CoV‐2, several strategies have already been tested to target it, for example, antibodies against ACE2 or pseudoligands to dominate the binding site for SARS‐CoV‐2.^55^ Moreover, in a recent study, a synthetic peptide homologous to the DPP4 receptor has been tested on three‐dimensional complex lung organoid structures derived from human‐induced pluripotent stem cells as immunotherapeutic candidates for COVID‐19 treatment.^56^

The aim of our pilot‐study is to verify if the deregulated expression of *ACE2* and *DPP4* genes, previously identified in COVID‐19 patients, can also be due to epigenetic mechanisms such as differential miRNA expression. Literature data described putative miRNAs that could target and regulate *ACE2* and *DPP4* genes.^29^, ^30^, ^31^, ^32^, ^33^ In particular, we investigated hsa‐let7b‐5p expression levels, which seems to target both the genes.^30^, ^31^

Our in silico analysis predicts the putative interaction of hsa‐let7b‐5p with both *ACE2* and *DPP4* mRNA. This analysis indicated *DPP4* and *ACE2* among hsa‐let7b‐5p putative target genes; in fact, both transcripts contained binding sites for hsa‐let7b‐5p (Table S1).

In vitro overexpression or inhibition of hsa‐let7b‐5p in an epithelial cell line (HeLa) showed a progressive increase in *ACE2* and *DPP4* mRNA levels after treatment with let7b‐5p inhibitor (Figure 1) and a significant decrease of transcripts levels after let7b‐5p mimic treatment (Figure 2).

Based on these data, we analysed the expression level of hsa‐let7b‐5p in naso‐oropharyngeal residual swabs of the same COVID‐19 patients and negative subjects for SARS‐CoV‐2 infection, analysed in our previous study.^21^ We revealed a statistically significant downregulation of hsa‐let7b‐5p in COVID‐19 patients (Figure 3). Accordingly, the expression level of hsa‐let7b‐5p showed a negative correlation with *ACE2* and *DPP4* expression level (Pearson *r* = −0.215 and −0.200, respectively) in NPS, although it does not reach statistical significance. The lack of statistical significance might be explained by the fact that hsa‐let7b‐5p is probably one of the factors that could regulate *ACE2* and *DPP4* expression in NPS, but probably it is not the only one. Moreover, the small number of samples could contribute to this result. We also analysed a possible correlation between the expression levels of hsa‐let7‐5p and any inflammatory markers or specific disease symptoms, but we did not observed any significant results.

The low expression level of hsa‐let7b‐5p that we observed in NPS of COVID‐19 patients is in agreement with previous studies, indicating its downregulation in patients with type 2 diabetes mellitus and in inflammation.^35^, ^36^, ^37^ Interestingly, a recent study suggested that, in aged COVID‐19 patients, a lower abundance of miRNAs might be a contributing factor in disease severity.^20^ Furthermore, six miRNAs (hsa‐miR‐1‐3p, hsa‐miR‐17‐5p, hsa‐miR‐199a‐3p, hsa‐miR‐429, hsa‐miR‐15a‐5p, and hsa‐miR‐20a‐5p) previously reported to be anti‐viral in respiratory diseases, were downregulated in lung tissues during viral infection but overexpressed in normal lung tissues.^13^

Moreover, we sequenced the *LET7B* gene in all 60 enrolled subjects and identified only one polymorphism in the *LET7B* gene, the rs143676966 (G > C). A not statistically significant association was observed between this polymorphism and the expression level of hsa‐let7b‐5p. This result seems to suggest that the genomic variability of this miRNA gene does not influence the expression levels of hsa‐let7b‐5p in NPS.

To the best of our knowledge, this is the first study that explores hsa‐let7b‐5p expression in naso‐oropharyngeal swabs of COVID‐19 patients, demonstrating a significant downregulation of this miRNA. Hsa‐let7b‐5p low expression in naso‐oropharyngeal cells of COVID‐19 patients may in part be correlated to the disease by means of a lack of regulation of genes (*ACE2* and *DPP4*) exploited by SARS‐CoV‐2. For this reason, we think that our results might be useful to scientific community and could provide the inspiration for further studies on miRNAs involvement in COVID‐19.

## AUTHOR CONTRIBUTIONS

**Andrea Latini:** Methodology (equal); writing – original draft (equal). **Chiara Vancheri:** Conceptualization (equal); investigation (equal); methodology (equal); writing – original draft (equal). **Francesca Amati:** Conceptualization (equal); data curation (equal); methodology (equal); writing – original draft (equal); writing – review and editing (equal). **Elena Morini:** Methodology (equal). **Sandro Grelli:** Resources (equal); validation (equal). **Matteucci Claudia:** Investigation (equal); validation (equal). **Petrone Vita:** Validation (equal). **Vito Luigi Colona:** Formal analysis (equal). **Michela Murdocca:** Methodology (equal). **Massimo Andreoni:** Resources (equal); supervision (equal). **Vincenzo Malagnino:** Investigation (equal). **Massimiliano Raponi:** Resources (equal); supervision (equal). **Dario Cocciadiferro:** Formal analysis (equal). **Antonio Novelli:** Supervision (equal). **Paola Borgiani:** Conceptualization (equal); data curation (equal); formal analysis (equal); writing – original draft (equal); writing – review and editing (equal). **Giuseppe Novelli:** Conceptualization (equal); funding acquisition (equal); project administration (equal); supervision (equal); writing – review and editing (equal).

## FUNDING INFORMATION

This study was supported in part by a grant of LazioInnova (Italy, Progetti di Gruppi di Ricerca 2020 A0375‐2020‐36663 GecoBiomark) to G.N. and A.N.

## CONFLICT OF INTEREST

The authors declare no conflict of interest.

## INFORMED CONSENT

Informed consent was obtained from all subjects involved in the study.

## Supporting information


Table S1

Table S2

## Data Availability

The data that support the findings of this study are available from the corresponding author upon reasonable request.
