# Supplementary material for: Expression analysis of miRNA hsa‐let7b‐5p in naso‐oropharyngeal swabs of COVID‐19 patients supports its role in regulating ACE2 and DPP4 receptors
Source: J Cell Mol Med. 2022 Sep 8;26(19):4940–8. doi: 10.1111/jcmm.17492 (PMC9538662; doi:10.1111/jcmm.17492)
Supplement: Supplementary file 1 — Table S1 [file JCMM-26-4940-s002.docx]

**Supplementary Table S1.** Hsa-let7b-5p binding site identified on coding DNA sequence of ACE2 and DPP4 genes.

| **Gene Symbol** | **Refseqid** | **Position** | **Binding Site Start-End** | **N Pairings** | **Score** |
| --- | --- | --- | --- | --- | --- |
|  |  |  |  |  |  |
| **ACE2** | NM_021804 | CDS | 1461-1482 | 17 | 1.00 |
|  |  |  | 402-423 | 15 | 0.92 |
|  |  |  | 717-741 | 17 | 0.90 |
|  | NM_001371415 | CDS | 1204-1225 | 17 | 1.00 |
|  |  |  | 145-166 | 15 | 0.92 |
| **DPP4** | NM_001935 | CDS | 1545-1571 | 19 | 0.85 |

MiRWalk online database was used to predict the binding sites between hsa-let7b-5p and the coding DNA sequence of ACE2 and DPP4 genes. CDS: coding sequence.
