# Supplementary material for: Expression analysis of miRNA hsa‐let7b‐5p in naso‐oropharyngeal swabs of COVID‐19 patients supports its role in regulating ACE2 and DPP4 receptors
Source: J Cell Mol Med. 2022 Sep 8;26(19):4940–8. doi: 10.1111/jcmm.17492 (PMC9538662; doi:10.1111/jcmm.17492)
Supplement: Supplementary file 2 — Table S2 [file JCMM-26-4940-s001.docx]

**Supplementary Table S2.** Clinical data of COVID-19 patients.

| **Characteristic** | **SARS-CoV-2 diagnostic test** |
| --- | --- |
|  | **Positive** |
| No. | 35 |
| Age | 62.0 ± 15.0 |
| Male (%) | 75 |
| Female (%) | 25 |
| Dry Cough (%) | 31 |
| Fever (%) | 66 |
| Dyspnea (%) | 57 |
| Gastrointestinal disorders (%) | 9 |
| FiO_2_ range (n, %) | N.A. (6, 17) |
|  |  |
|  | 35 – 50 (12, 34) |
|  | 35 – 80 (2, 6) |
|  | 50 – 60 (5, 14) |
|  | 50 – 80 (2, 6) |
|  | 60 – 80 (3, 9) |
|  | 80 – 100 (1, 3) |
| Hypertension (%) | 34 |
| Neu (10^3^/µL) | 5.2 ± 3.3 |
|  | (*neutrophilia in 7/35 patients: 9.9 ± 4.6) |
| Lym (10^3^/µL) | 1.3 ± 0.7 |
|  | (*important lymphopenia in 23/35 patients: 0.9 ± 0.4) |
| CRP (mg/L) | 67.2 ± 75.2 |
|  | (*elevated values in 25/35 patients: 93.1 ± 74.5) |
| LDH (U/L) | 364.1 ± 192.2 |
|  | (*elevated values in 25/35 patients: 407.8 ± 189.0) |
| TNF-α (pg/ml) | 31.8 ± 36.7 |
| IL-6 (gr/dl) | 40.9 ± 46.6 |
| Fibrinogen (mg/dl) | 573.7 ± 198.4 |
| D-dimer (ng/mL) | 3083.4 ± 7088.3 |

Continuous data are expressed as mean ± standard deviation (SD); categorical data are expressed as percentage (%), n (number of cases). FiO_2_ (Fraction of inspired oxygen). Neu (neutrophils; normal values 1,5-7 103/mL). Lym (lymphocyte; normal values 1,5-5 103/mL). CRP (C-Reactive Protein; normal values 0-5 mg/L). LDH (Lactate dehydrogenase; normal values 125-220 U/L). TNF-α (Tumor Necrosis Factor alfa; normal values <50 pg/ml). IL-6 (Interleukin-6; normal range values = 4.6 - 12.4; low <4.6; high> 12.4). Fibrinogen (normal values 200- 400 mg/dl). D-dimer (normal values 0-500 ng/ml).
